# Haute prévalence du Burnout dans les unités Tunisiennes prenant en charge des patients en fin de vie

**DOI:** 10.11604/pamj.2014.19.9.2865

**Published:** 2014-09-04

**Authors:** Badii Amamou, Ahmed Souhaiel Bannour, Meriem Ben Hadj Yahia, Selma Ben Nasr, Bechir Ben Hadj Ali

**Affiliations:** 1 Service de psychiatrie, CHU Farhat Hached de Sousse, Faculté de Médecine Ibn Jazzar de Sousse, Université de Sousse, Tunisie

**Keywords:** Burnout, infirmiers, soins de fin de vie, Tunisie, Burnout, nurses, end of life care, Tunisia

## Abstract

**Introduction:**

Chez le personnel soignant, le burnout touche un infirmier sur trois. Ce taux est plus élevé dans les unités prenant en charge des patients en fin de vie. L'objectif de notre travail était d'évaluer la fréquence du burnout chez les infirmiers qui travaillent en soins de fin de vie.

**Méthodes:**

Il s'agit d'une étude descriptive transversale réalisée entre le 1er Avril et le 31 Mai 2010. 60 infirmiers de six services de Sousse et de Monastir (Tunisie) ont été recrutés. L'évaluation du burnout a été réalisée par deux échelles: MBI (Maslach Burnout Inventory) et BMS (Burnout Measure Short version).

**Résultats:**

La prévalence du burnout était de 70%; il était élevé chez 81.7%. 80% avaient un niveau élevé d'épuisement émotionnel, 70% avaient un niveau élevé de dépersonnalisation et 17% avaient un niveau bas de sentiment d'accomplissement personnel. Le burnout était plus élevé chez les hommes (70,8% vs 69,4%; p=0,013); ceux qui voulaient améliorer les conditions du travail (70.2% vs. 66.7%; p= 0.017); du salaire (70.2% vs. 66.7%; p= 0.017) et chez les infirmiers suivi en psychiatrie (71.4% vs. 69.8%; p= 0.008).

**Conclusion:**

Dans notre étude le niveau de burnout était élevé chez les infirmiers prenant en charge des patients en fin de vie. Il était associé au sexe masculin et à l'insatisfaction des conditions de travail et du salaire. D'autres études longitudinales sont nécessaires pour suivre l'évolution de ce syndrome et mettre des stratégies de prévention adéquates.

## Introduction

Les premières publications concernant le burnout sont parues au milieu des années 70 dans le cadre de la désinstitutionalisation des soins aux USA [1]. Le burnout ou épuisement professionnel est défini selon l'OMS comme « un sentiment de fatigue intense, de perte de contrôle et d'incapacité à aboutir à des résultats concrets au travail ». Trois symptômes cardinaux définissent l'état de burnout: l'épuisement émotionnel, la dépersonnalisation et l'échec de l'accomplissement personnel [2]. Maslach affirme que le syndrome d'épuisement professionnel est un processus qui provient d'un déséquilibre entre les exigences du travail et les ressources de l'individu. Ces dernières peuvent être personnelles (estime de soi, auto efficacité…) ou organisationnelles (soutien reçu de la part des collègues, de la hiérarchie…). Le burnout résulte donc d'un épuisement des capacités d'adaptation de l'individu [2].

Plusieurs études épidémiologiques se sont intéressées à évaluer l'ampleur du burnout en milieu professionnel. Les taux de prévalence variaient de 25 à 60% chez le personnel soignant [3, 4], le burnout touche un infirmier sur trois, selon l'association américaine de recherche en soins infirmiers [5, 6]. Des recherches sur le personnel infirmier au Canada, aux Etats Unis et en Angleterre avaient révélé que 32,9% à 54,2% avaient un burnout [5–7]. Dans les pays en voie de développement le manque d′effectifs et de moyens, ainsi que le salaire bas seraient à l'origine d'une souffrance plus marquée [8, 9]. Dans les pays arabes en voie de développement le concept d'épuisement professionnel n'est pas encore bien exploré. Toutefois, les études menées dans quelques pays comme la Jordanie, la Turquie et l'Arabie Saoudite avaient montré que les infirmiers, dans ces pays, les taux de burnout pouvaient dépasser les 50%. [5, 10–12]. En Tunisie, pays qui se rapproche culturellement de ces derniers mais ayant un système de soins totalement différent, une étude réalisée auprès de 54 infirmiers en milieu psychiatrique avait montré que 35,8% d'entre eux souffraient d'épuisement professionnel [13]. Les études récentes indiquent que certaines composantes de l'organisation du travail comme l'instabilité des équipes [7, 10], l'absentéisme qui se multiplie, les tâches multiples de l'infirmier, les horaires difficiles, les difficultés de conciliation travail-famille, le contact constant avec la souffrance et avec la mort pourraient contribuer au développement du burnout [14–21].

Les études qui se sont intéressées aux infirmiers travaillants dans les unités de carcinologie, d'hématologie, des urgences et de la réanimation subissent divers types de stress (physique, émotionnel, cognitif, organisationnel…) et seraient plus sujets à l'épuisement professionnel [21–26]. Ainsi, les professionnels de soins qui s'occupent de patients en fin de vie semblent subir des facteurs de stress plus important pouvant conduire à des taux élevés d'épuisement professionnel. [27, 28] L'objectif de notre travail était d'évaluer la fréquence de l'épuisement professionnel chez les infirmiers qui travaillent en soins de fin de vie et de déterminer les facteurs qui y sont associés.

## Méthodes

### Echantillon de l'étude

Il s'agit d'une étude descriptive transversale réalisée pendant les mois d'Avril et Mai 2010, dans les hôpitaux Sahloul et Farhat Hached de Sousse et dans l'hôpital Fattouma Bourguiba de Monastir, en Tunisie. La population cible est constituée de tous les infirmiers exerçant dans deux services des urgences, un service de réanimation médicale, un service de réanimation chirurgical, un service d'hématologie et un service de carcinologie. Ces services, de soins intensifs et palliatifs, exposent fréquemment les infirmiers à des soins de fin de vie. Le choix de ces services a été basé sur le fait qu'ils sont des unités de soins intensifs et palliatifs, où les intervenants sont fréquemment exposés à assurer des soins de fin de vie. Ainsi, les services de réanimation, d'hématologie et de carcinologie assurent des soins palliatifs pour des patients au stade terminal de leurs maladies. Les services des urgences constituent le seul recours de ces patients en cas de nécessité de soins urgents et y reviennent souvent de façon récurrente. En effet, aussi bien à Sousse qu'à Monastir, il n'y a pas d'unités spécialisées dans les soins de fin de vie qui sont coordonnés par les services de médecine, les urgences et les services de réanimation. les études antérieures [22–24] ayant procédé à une évaluation des soins de fin de vie ont étés réalisés dans des unités de soins intensifs exposants le personnel infirmier aux soins intensifs et palliatifs. 60 infirmiers, parmi 129, ont accepté de participer à l'étude, ce qui fait un taux de participation de 46,5%.

### Instruments d'évaluation

#### L'évaluation du burnout a été réalisée par deux échelles

L'échelle MBI (Maslach Burnout Inventory) qui caractérise le burnout et permet d'explorer ses 3 dimensions: l'épuisement émotionnel (exploré par 9 items), la dépersonnalisation (explorée par 5 items) et l'accomplissement personnel (exploré par 8 items). [2] C'est l'échelle la plus utilisée dans la littérature pour décrire chaque dimension du burnout en milieu professionnel. L'échelle BMS (Burnout Measure Short version) composée de 10 questions et qui représente une version courte de l'échelle MBI. Comparativement à la MBI, elle permet de restituer un score unique d'épuisement physique, mental et émotionnel et d'évaluer ainsi le degré de burnout. Elle permet, de ce fait, une comparaison entre des sujets présentant un épuisement et ceux sans épuisement. [28] Un auto-questionnaire anonyme, formé de 29 questions, a été élaboré pour le recueil: des caractéristiques sociodémographiques et professionnelles des sujets interrogées: 8 questions; des facteurs de risque du burnout: 17 questions; des solutions proposées, par le personnel infirmier, pour prévenir ou gérer le syndrome du burnout: 4 questions.

#### Déroulement de l'étude

Des autorisations de la part des chefs des services et des surveillants des services concernés par notre étude ont été prises avant le début de l'étude. Les questionnaires ont été distribués aux infirmiers pendant l'exercice de leur activité professionnelle et ce, en tenant compte de la répartition des horaires de travail dans leurs services. Un consentement libre et éclairé a été déclaré de la part des infirmiers interrogés après leurs avoir expliqué clairement les objectifs de notre travail de recherche.

#### Analyse des données

Les données ont été analysées par le logiciel SPSS version 17. Notre travail a consisté en premier lieu en une étude descriptive des caractéristiques sociodémographiques de la population. Puis, des corrélations entre les scores du burnout et les différentes caractéristiques sociodémographiques ont été effectuées en utilisant le test de Pearson. Les résultats ont été considérés significatifs pour une valeur de p<0,05.

## Résultats

### Caractéristiques sociodémographiques et professionnelles

60% de notre échantillon étaient des femmes et 40% des hommes. Le sexe ratio homme/femme était de 0,66. 60% de notre échantillon étaient âgés de 20 à 30 ans. 58,3% étaient mariés et 38,3% étaient célibataires. 98% des infirmiers mariés avaient moins de 4 enfants à leur charge. 13,3% des infirmiers interrogés exerçaient depuis plus de 20 ans et 63,3% avaient une ancienneté dans la fonction inférieure à 5 ans. 91,7% des sujets de notre étude avaient le grade d'infirmier et 88,3% la fonction d'infirmier. (Tableau 1)

**Tableau 1:** Caractéristiques sociodémographiques et professionnelles de l'échantillon

Modalité		Effectif (n)	Pourcentage (%)
**Age**	20 -40 ans	51	85
	Plus de 40 ans	9	15
**Sexe**	Masculin	24	40
	Féminin	36	60
**Etat civil**	Marié	35	58,3
	Célibataire	23	38,3
	Divorcé	2	3,3
	Veuf	0	0
**Ancienneté dans le service**	0-5 ans	38	63,3
	6-10 ans	7	11,7
	11-15 ans	4	6,7
	16-20 ans	3	5
	Plus de 20ans	8	13,3
**Fonction**	Infirmier(e)	53	88,3
	Chef d'équipe	3	5
	Surveillant	4	6,7
**Répartition des infirmiers selon les services choisis**	Réanimation Médicale FH	15	25
	Réanimation Médicale Monastir	12	20
	Réanimation Chirurgicale Sahloul	7	11,7
	Urgence FH	7	11,7
	Hématologie FH	10	16,7
	Carcinologie FH	9	15

### Fréquence des facteurs de risque du burnout

**Facteurs organisationnels:** 88,3% des infirmiers avaient vécu un stress durable au travail. 68,3% avaient confirmé l'absence de soutien de la hiérarchie. 65% des infirmiers avaient rapporté l'absence d'écoute dans leur service et 71,7% l'absence de groupes de paroles. 95% des infirmiers avaient confirmé la nécessité d'améliorer les conditions de travail dans leurs services.

**Facteurs professionnels:** 91,7% des infirmiers n'avaient pas bénéficié d'une formation spécifique concernant la prise en charge des sujets en fin de vie. 60% de notre échantillon n'étaient pas impliqués dans les décisions thérapeutiques de l'équipe soignante. 25% avaient vécu des doutes en leurs capacités professionnels à faire face à une situation de fin de vie pénible. 88,3% des infirmiers n'avaient jamais reçu de marques de reconnaissances de la part des patients ou des remarques gratifiantes d'un supérieur dans leur travail.

**Facteurs personnels:** 92,3% des infirmiers avaient des conflits avec des membres de leur équipe soignante. 60% n'avaient pas choisi le service dans lequel ils travaillent actuellement et 78,3% avaient des difficultés relationnelles avec les patients en fin de vie. 28,3% des infirmiers n'avait jamais pratiqué une activité physique. Sept (11,7%) avaient été suivis pour un problème de santé mentale.

### Prévalence du burnout

**Echelle de BMS:** 70% des infirmiers interrogés souffraient d'un syndrome de burnout. 63,3% avaient un burnout modéré, 3,4% avaient un burnout très élevé et 3,3% avaient un burnout nécessitant une intervention. (Tableue 2)

**Tableau 2:** Prévalence du burnout et de ses trois dimensions

		Effectif	Pourcentage (%)
**Absscence de burnout (BMS)**		18	30
**Présence de burnout (BMS)**	Modéré	38	63.3
	Très élevé	2	3.4
	Nécessitant une intervention	2	3.3
**épuisement émotionnel(MBI)**	Bas	6	10
	Modéré	5	8.3
	Elevé	49	81.7
**Dépersonnalisation(MBI)**	Bas	7	11.7
	Modéré	11	18.3
	Elevé	42	70
**Accomplissement personnel(MBI)**	Bas	10	16.7
	Modéré	25	41.7
	Elevé	25	41.6

**Echelle de MBI:** 81,7% des infirmiers interrogés avaient un épuisement émotionnel élevé et 70% avaient un niveau de dépersonnalisation élevé. L'accomplissement personnel était bas chez 16,7% des infirmiers alors qu'il était modéré et/ou élevé chez 83,3% de la population. (Tableau 2)

### Facteurs associés au burnout

La fréquence du burnout était significativement plus élevée chez les sujets de sexe masculin comparativement à ceux de sexe féminin (70,8% vs 69,4%; p=0,013). Nous n'avons pas noté d'association entre le burnout et l’âge, le statut marital, l'ancienneté dans la fonction, l'ancienneté dans le service, l'existence d'une formation spécifique, les doutes en ses capacités professionnels et les reconnaissances. La fréquence du burnout étaient significativement plus élevée chez les infirmiers qui estiment une incompatibilité entre leurs salaires et la charge du travail comparativement à ceux n'estimant pas l'existence d'une incompatibilité (66,7% vs 70,2% p= 0,017).

Nous n'avons pas noté d'association entre le burnout et la participation aux décisions thérapeutiques de l'équipe soignante, l'écoute au sein du service, le soutien de la hiérarchie et les groupes de parole. La prévalence du burnout était significativement plus élevée chez les sujets qui voulaient améliorer les conditions du travail comparativement à ceux qui s'en estimaient satisfaits (70,2% vs 66,7% p=0,017). Nous n'avons pas noté d'association entre le burnout et l'existence d'un stress durable au travail, de conflits professionnels et de difficultés relationnelles avec les patients en fin de vie. La pratique d'une activité physique n'a pas été associée au burnout. La prévalence du burnout était significativement plus élevée chez les infirmiers suivis en psychiatrie comparativement à ceux non suivis (71.4% vs 69.8%; p= 0.008). (Tableau 3).

**Tableau 3:** Facteurs associés au burnout

		Modalité	Prévalence du burnout (%)	Corrélation: p
**facteurs sociodémographiques**	Sexe	Masculin	70.8	p=0,013
		Feminin	69.4	
	Age	<40 ans	70.6	p=0,056
		>40 ans	66.7	
	Statut marital	Marié	86.6	p=0,082
		Non marié	72	
**facteurs organisationnels**	Stress	Oui	73,6	p= 0.182
		Non	42,9	
	Ecoute au service	Oui	61,9	p=0.381
		Non	74,4	
	soutien de la hiérarchie	Oui	63,2	p= 0,620
		Non	73,2	
	Groupes de parole	Oui	76,5	p= 0.550
		Non	67,4	
**Facteurs professionnels**	Ancienneté dans le service	Moins de 10 ans	71,1	p=0.887
		Plus de 10 ans	66,7	
	Formation spécifique	Oui	20	p=0.025
		Non	74,5	
	Doute en ses capacités professionnelles	Oui	73,3	p=0,106
		Non	68,9	
	Reconnaissance	Toujours	28,6	p=0.212
		Jamais	78	
	salaire et charge infirmière	**Compatible**	**66.7**	**p=0,017**
		**incompatible**	**70.2**	
	Condition de travail	Satisfaisantes	66.7	**p=0,017**
		A améliorer	70.2	
**Facteurs personnels**	Conflits	Oui	72	p=0.468
		Non	60	
	Difficultés relationelles	Oui	74.5	p=0.181
		Non	53.8	
	Activité sportive	Oui	61.5	p=0,922
		Non	72.3	
	Problème de santé psychiatrique	**Oui**	**71.4**	**p= 0,008**
		**Non**	**69.8**	
	La possibilité de prendre un congé quand il/elle le désire	Oui	67.7	p=0,156
		Non	72.4	
	Choix du service	Oui	58.3	p= 0.152
		Non	77.8	

### Solutions envisagées par le personnel

78,7% des infirmiers désiraient avoir une formation spécialisée sur le deuil et l'accompagnement des patients en fin de vie. 83,3% pensaient qu'un soutien extérieur leurs serait bénéfique. Parmi les solutions proposées pour gérer leur épuisement professionnel un personnel médical spécialisé était proposé.

## Discussion

L'épuisement professionnel est une réalité chez les infirmiers travaillants dans les services de soins de fin de vie où ils étaient exposés à une charge infirmière marquée par un stress professionnel important. Dans notre étude la prévalence du burnout était de 70%. 81,7% des infirmiers présentaient un épuisement émotionnel élevé, 70% avaient un niveau de dépersonnalisation élevé et 16,7% avaient un niveau d'accomplissement personnel bas.

La majorité des études épidémiologiques avaient rapporté des syndromes du burnout sévères chez 25 à 60% des professionnels de santé [29–32]. Des recherches sur le personnel infirmier au Canada, aux Etats Unis et en Angleterre avaient révélé que 38,3 à 48,1% des infirmiers avaient déclaré être insatisfaits au travail et que 32,9 à 54,2% avaient un burnout [5, 7]. Dans les pays arabes le concept d'épuisement professionnel n'est pas encore bien exploré. Toutefois, les études menées dans quelques pays comme l'Algérie et l'Arabie Saoudite avaient montré que les infirmiers, dans ces pays, soufraient bien de l'épuisement professionnel [5, 10]. Une étude réalisé en Jordanie sur 181 infirmiers en psychiatrie a trouvé 32.7% d'épuisement émotionnel, 27.7% de dépersonnalisation et 16.8% d'échec de l'accomplissement personnel [11]. Une étude turque réalisée aux services des urgence incluant 76 infirmiers a trouvé 53% d'épuisement émotionnel, 39% de dépersonnalisation et 46% d'échec de l'accomplissement personnel sans relation avec l'état matrimonial travail, salaire satisfaction sauf pour la satisfaction profesionelle [12]. En Tunisie, il y a eu peu d'études sur le burnout. Les seuls résultats dont nous disposons sont celles publiés par Halayem, Dhouib et al. qui avaient trouvé un taux d'épuisement professionnel de 35,8% [13]

Le taux de prévalence élevé observé dans notre échantillon serait lié au fait que les infirmiers sont exposés à des situations de fin de vie et par conséquent à des facteurs de risque plus important de burnout. Les travaux réalisés dans des unités de soins intensifs, des urgences de carcinolgie et d'hématologie avaient révélé que la prise en charge d'un patient agonisant accroîtrait le risque de souffrir d'un syndrome de burnout [20, 21, 24, 25]. Alors que pour Pereira le niveau de burout ne semblait pas augmenter chez les infirmiers travaillant dans les soins palliatifs. [33] Dans notre étude le sexe masculin était associé au burnout (70,8% vs 69,4%; p=0,013). Les résultats de la littérature sont sujets de controverses. Catts et al. [34] ont trouvé le même résultat dans une population de 39 infirmiers exerçant en carcinologie. Ils ont incriminé la charge du travail plus importante donnée au sexe masculin. L'équipe de Kovacs avait trouvé une corrélation entre dépersonnalisation et sexe masculin [14]. Les équipes de Poncet, Di Iorio et Raggio ont par contre trouvé que les infirmiers de sexe féminin étaient plus touchés par ce syndrome que leurs collègues de sexe masculin [26, 35, 36]. Les explications avancées étaient que les femmes avaient une plus importante capacité d'introspection par rapport aux hommes. Dans notre travail l’âge et le statut marital n'étaient pas corrélés au burnout. Les équipes de Grau, Hanrahan, Trindadelde et Kim avaient rapporté que les jeunes infirmières semblaient être plus touchées par cette symptomatologie [37–40]. De plus, Moreira et al. avaient trouvé une association entre burnout et le fait d’être une femme jeune âgée de 26 à 35 ans, mariée, sans enfants et travaillant depuis plus de cinq ans [8].

La durée d'exercice professionnel, non corrélée au burnout dans notre étude, reste sujet de controverses. En effet, plusieurs auteurs ont trouvé que le burnout était corrélé négativement à l'expérience professionnelle [41, 42]. Cependant, d'autres auteurs avaient affirmé que le burnout était plus prévalent chez les infirmiers qui ont plus d'expérience professionnelle. Montero précise que le burnout était significativement plus fréquent chez les infirmiers exerçant depuis plus de seize ans [41]. Les infirmiers estimant qu'il existe une incompatibilité entre leurs salaires et leurs charges de travail avaient un risque plus élevé du burnout que ceux qui étaient satisfaits de leurs salaires.

Nous n'avons pas trouvé de corrélation significative entre la reconnaissance des patients ou l'encouragement d'un cadre supérieur dans le travail et le burnout. Cependant Vimantaite et Vahey avaient affirmé qu'un patient qui exprime une reconnaissance aux soignants, contribuant à sa guérison ou à son soutien moral, renforçait la personnalité et protégeait d'une souffrance souvent inconsciente chez le soignant [43, 44]. Dans notre étude le taux de prévalence du burnout chez les infirmiers non satisfaits des conditions du travail était de 70,2%. Ce résultat significativement corrélé au burnout rejoint les résultats trouvés dans la littérature [45] où plusieurs auteurs accusent la grosse charge et les mauvaises conditions de travail [30, 46] d’être à l'origine du burnout. Une étude réalisée en Afrique du sud a trouvé que le burnout était lié à l′insatisfaction des salaires, des possibilités d′avancement, au manque de personnel et de ressources, ainsi qu'à la faible participation des infirmiers dans les décisions hospitalières [47]. Rappelons que le personnel interrogé, dans notre travail, se plaignait de l'insécurité au travail, du manque de personnel, de l'étroitesse des locaux et du bruit. L'équipe de Gulalp quand a elle n'avait pas trouvé de relation entre burnout et condition de travail (charge, horaire, salaire) [48].

Dans notre étude l'écoute au service et le soutien de la hiérarchie n'étaient pas associés au burnout. Toutes les enquêtes ergonomiques avaient montré des effets modérateurs du soutien sur le stress perçu. De plus, la plus part des auteurs défendaient l'idée que l'écoute au sein du service serait un facteur protecteur du burnout avec une corrélation significative entre l'insuffisance d'écoute au sein du service et l'épuisement professionnel [44, 49]. L'absence d'une formation à la communication chez les infirmiers des services étudiés serait à l'origine d'un manque de structuration de l'écoute et du soutien. Ainsi, même si les infirmiers se sentent suffisamment soutenus ou écoutés ceci ne semble pas les protéger de l'épuisement professionnel. Shimizu avait trouvé une baisse significative du burnout après une formation à la communication avec une meilleure négociation et une acceptation des critiques [50]. Dans notre étude, il n'y avait pas d'association entre le stress et le burnout. Alors que l'ensemble des auteurs s'accordait à dire que le burnout aurait des origines externes, liées à la situation de stress. [51] Certains mettent en cause dans le travail d'infirmier l'ambiguïté′ des rôles [32, 44]. Cela est doublement le cas ici ou‘ s'ajoutent les difficultés spécifiques du travail dans les services de soins de fin de vie: la relation difficile avec des patients dont le pronostic est réservé [25]. Nous n'avions pas trouvé d'association entre la confrontation à la souffrance du patient et aux difficultés relationnelles lors d'une situation de fin de vie et le burnout. Dans une étude réalisée sur 272 infirmiers aux services des urgences en Roumanie, Popa avait trouvé qu'il existait une relation statistiquement significative entre le burnout et la confrontation à la souffrance du patient et aux difficultés relationnelles. [42] La confrontation avec la souffrance est quelque chose qui peut affecter la journée entière du travail dans certains types de soins et qui de ce fait nécessite une adaptation constante. En effet, Maslach considérait le fait de ne plus penser aux patients et à leurs problèmes après le travail comme un facteur de réduction du stress [2].

Concernant les conflits entre les membres de l'équipe soignante, les équipes de Azoulay et Lorenz ont noté une relation entre les conflits professionnels et la survenu du burnout [49, 51]. Il a été démontré que le fait de travailler dans une ambiance tendue, sous un stress constant était un facteur qui favorisait les conflits entre le personnel. Ces conflits au sein des collègues et des médecins étaient corrélés à un burnout accru. Dans notre étude l'existence d'une maladie psychiatrique était associée au burnout. Catt avait trouvé une comorbidité psychiatrique probable de 5 à 27% avec le burnout chez 10 équipes de carcinologie en Angleterre [52]. Dionisios avait étudié la relation entre alexithymie (incapacité à reconnaitre et à exprimer les émotions) et burnout. Il avait trouvé une corrélation avec la dépression, l'épuisement professionnel et la dépersonnalisation [53]. Kovacs avait, en outre, trouvé que certains troubles de contrôle des émotions, surtout négatives, pouvaient être décisifs dans l'émergence du burnout [54]. Rafii quand à lui avait démontré que l'interaction des personnalités du patient et de l'infirmier était à l'origine du burnout en altérant les émotions, les attitudes, le comportement et l'organisation des soins [55]. En étudiant les traits de personnalité associés au burnout chez 707 infirmières au japon, Shimizutani avait trouvé une corrélation entre névrose et burnout.

### Limites

Le faible taux de participation des infirmiers: 46,5% (60 infirmiers parmi 129 ont participés) constitue une des limites de cette étude et comme le questionnaire de Maslasch peut susciter de la culpabilité le taux de burnout pourrait être sous-estimé. Une seconde limite de cette étude est la prédominance féminine (60% de l'échantillon) qui pourrait surestimer les résultats vu que la majorité des auteurs s'accordent à dire que les infirmiers de sexe féminin sont plus exposés au burnout. L’âge jeune (85% étaient dans la tranche 20-40 ans) et l'expérience inférieure à 5 ans chez 63,3% de la population peuvent être considérés comme une autre limite de notre étude et pourrait surestimer le taux de burnout vu que ces deux conditions ont été corrélés au burnout dans plusieurs études. De plus nous n'avons utilisés que des auto-questionnaires ce qui rendrait les réponses «subjectives» et nous n'avons pas fait de mesure «objective» de la dépression. Enfin, une étude longitudinale pourrait amener des renseignements supplémentaires quant à la stabilité des symptômes dans le temps.

## Conclusion

Dans notre étude, le niveau de l'épuisement professionnel était élevé. Il était plus marqué chez les infirmiers de sexe masculin et chez ceux qui étaient insatisfaits des conditions de leur travail et de leur salaire. D'autres études longitudinales seraient nécessaires pour pouvoir suivre l'évolution de ce syndrome et mettre ainsi des stratégies de prévention et de prise en charge adéquates.
